# Identification of COM Controller of a Human in Stance Based on Motion Measurement and Phase-Space Analysis

**DOI:** 10.3389/frobt.2021.729575

**Published:** 2022-01-04

**Authors:** Tomomichi Sugihara, Daishi Kaneta, Nobuyuki Murai

**Affiliations:** ^1^ OMRON Corporation, Tokyo, Japan; ^2^ Kawada Robotics Corporation, Tokyo, Japan

**Keywords:** standing stabilization, human motor control, COM-ZMP regulator, system identification, piecewise-affine dynamical system

## Abstract

This article proposes a process to identify the standing stabilizer, namely, the controller in humans to keep upright posture stable against perturbations. We model the controller as a piecewise-linear feedback system, where the state of the center of mass (COM) is regulated by coordinating the whole body so as to locate the zero-moment point (ZMP) at the desired position. This was developed for humanoid robots and is possibly able to elaborate the fundamental control scheme used by humans to stabilize themselves. Difficulties lie on how to collect motion trajectories in a wide area of the state space for reliable identification and how to identify the piecewise-affine dynamical system. For the former problem, a motion measurement protocol is devised based on the theoretical phase portrait of the system. Regarding the latter problem, some clustering techniques including *K*-means method and EM (Expectation-and-Maximization) algorithm were examined. We found that a modified *K*-means method produced the most accurate result in this study. The method was applied to the identification of a lateral standing controller of a human subject. The result of the identification quantitatively supported a hypothesis that the COM-ZMP regulator reasonably models the human’s controller when deviations of the angular momentum about the COM are limited.

## 1 Introduction

Standing stabilization control, namely, the control to keep upright posture stable against perturbations, is one of the fundamental functions in humans and has been studied, through which the ankle and hip strategies have been hypothesized (Nashner and McCollum, 1985; Mueller et al., 1994; Gatev et al., 1999; Vette et al., 2007). The discussions mainly focused on a coordination of a couple of joints in the sagittal plane under specific conditions in the beginning, and has been extended to a control scheme in more generalized situations at various postures against various types of perturbations, where the lower and upper bodies move in-phase in the lower frequency domain and counter-phase in the higher domain (Goodworth and Peterka, 2010). Some works to implement the ankle/hip strategies on humanoid robots (Atkeson and Stephens, 2007; Lippi et al., 2016) have also been made.

How humans synthesize behaviors on their complex musculo-skeletal system into such control strategies has been of great interest to researchers in the field of body science and studied in depth for decades. Advanced technology has enabled precise motion measurements and a large-scale simulation of a detailed neuro-musculo-skeletal model of a human (Murai et al., 2008). However, the identification of human’s motor controller is still challenging. On the other hand, comprehensive studies of the whole-body dynamics have been made in the field of humanoid robotics, where some key aspects such as floating-base dynamics (Yoshida et al., 1995; Fujimoto and Kawamura, 1998) and the structure-varying nature (Nakamura and Yamane, 2000) have been discussed. It was also shown to be effective to focus on the macroscopic relationship between the center of mass (COM) and the center of pressure (COP), to which the zero-moment point (ZMP) (Vukobratović and Stepanenko, 1972) was given as an alias in the context of the motion synthesis. Although it omits the complex control that exploits counter-phase movements of the upper body, it plays a fundamental role in the stabilization in the reduced-order dynamics that can be visually analyzed in the phase space. Several controllers for humanoid robots were developed based on it (Mitobe et al., 2000; Sugihara et al., 2002; Kajita et al., 2003; Morimoto et al., 2008; Sugihara, 2009) In particular, a control scheme to regulate the COM by manipulating the ZMP was discussed (Sugihara, 2009). This enabled an intuitive understanding of the relationship between the response to perturbations and the stability performance under the limited supporting region compared with another scheme to manipulate the distribution of ground reaction forces (Hyon, 2009; Henze et al., 2016). It was also found to be related to the extrapolated center of mass (XCOM) (Hof, 2008) studied in the field of biomechanics. This suggests a possible hypothesis that human’s standing controller can be modeled by the COM-ZMP regulator. A study to compare it with the humans’ standing controller was also made (Peterka, 2009).

A problem that arises when applying the system identification to the human controller based on the model is that it is difficult to collect a sufficient number of motion trajectories since humans in general unconsciously stabilize themselves and hardly show behaviors in a distance from the point of equilibrium. A particular protocol to observe such behaviors has to be devised for this purpose. Another difficulty is that the system is piecewise, namely, the state space of the COM is divided into some regions described by different equations of motions due to the unilaterality of contact forces. It is a chicken-and-egg problem to identify such a system since the equation of description has to be provided to identify system parameters, while the system parameters are required to choose the equation of description (Bemporad et al., 2003; Ferrari-Trecate et al., 2003; Vidal et al., 2003).

The contributions of this article are twofold. First, we propose a method to collect motion trajectories in a wide area of the state space. The behaviors to be observed in each region can be predicted based on the dynamics of the COM-ZMP regulator in a phase portrait. Then, we add preparatory motion to each trial to accelerate the COM to preferable initial states. The preparatory phase in each motion trajectory is detected and discarded in the data processing based on the profile of the ground reaction force. Second, we propose methods to identify parameters of the piecewise system from the measured motion data. We examined *K*-means method (MacQueen, 1967; Duda and Hart, 1973) and EM (Expectation-and-Maximization) algorithm (Dempster et al., 1977) to cluster points in the state space into consistent regions and identify the system parameters in an iterative way. It was verified that both methods successfully output consistent results of the identification and a modified *K*-means method produced the most accurate result in this study. The entire process in which the above methods were combined was applied to the stabilization motion in the lateral direction of a human subject. Although the number of subjects was only one, the result supported the hypothesis that the human’s behavior can be modeled by the COM-ZMP regulator when deviations of the angular momentum about the COM are limited and thereby the translational movement of the COM is dominant. To the best of the authors’ knowledge, this is the first work that identified the standing controller of a human based on the model. Notice that the objective of this article is to propose the process, and to validate the hypothesis based on a number of results is outside the scope of this article.

Earlier versions of this work were presented at 2012 IEEE-RAS International Conference on Humanoid Robots (Kaneta et al., 2012), 2013 IEEE/RSJ International Conference on Intelligent Robots and Systems (Kaneta et al., 2013; Murai et al., 2013), and The 8th IEEE RAS/EMBS International Conference on Biomedical Robotics and Biomechatronics (Murai and Sugihara, 2020).

## 2 Dynamics Model of the COM-ZMP Regulator

**FIGURE 1:**
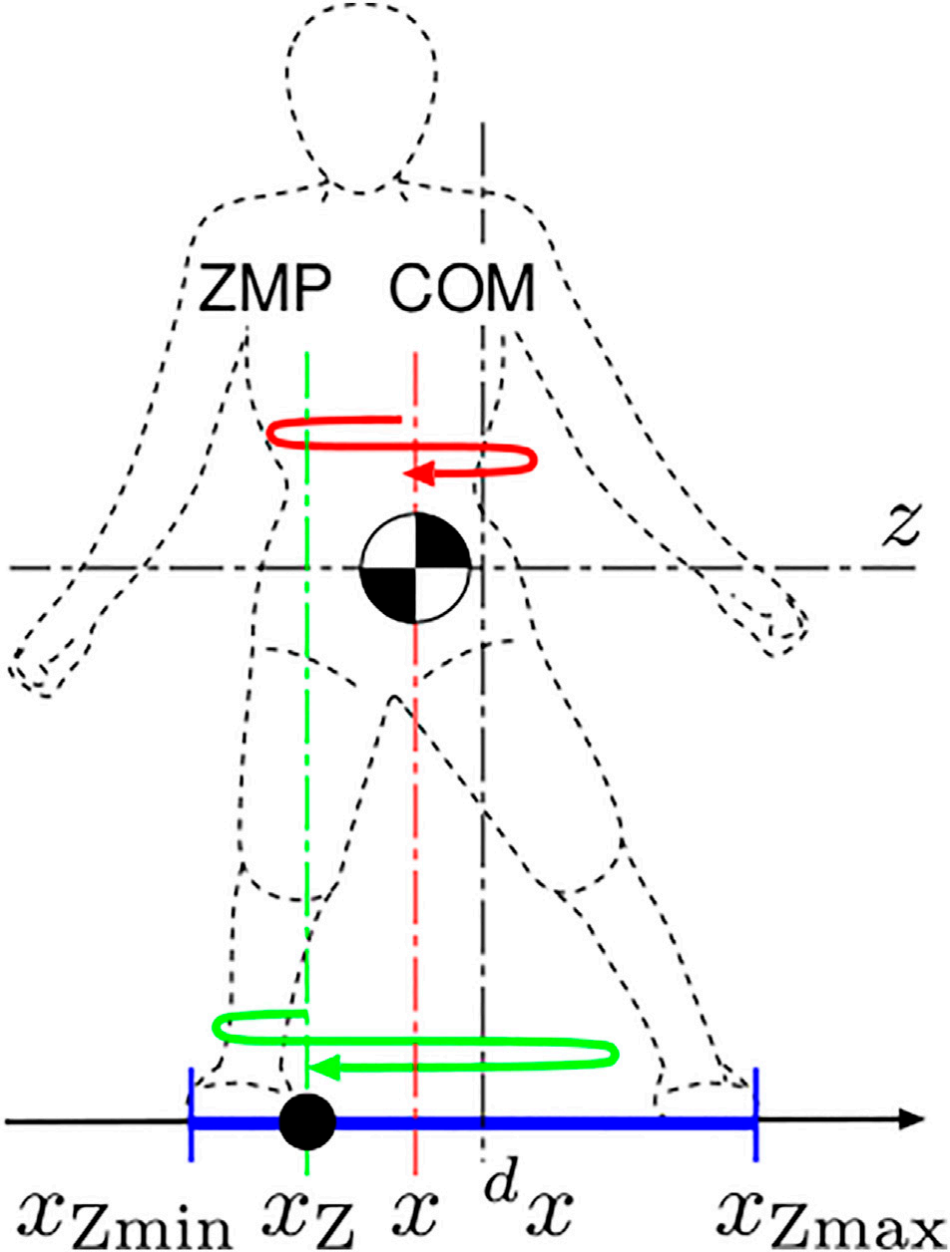
The COM-ZMP model of lateral standing motion. *z* is the height of the COM with respect to the nominal ground, *x* is the lateral position of the COM, ^*d*^ *x* is the referential position of the COM, *x*_Z_ is the lateral position of the ZMP, and *x*_Zmin_ and *x*_Zmax_ are the minimum and maximum boundaries of the supporting region, respectively.

The dynamics of a humanoid, which could be either a real human or a humanoid robot, is represented by a large-scale equation of motion and many inequalities originated from the limitation of contact forces. However, macroscopic characteristics embedded in the dynamics can be abstracted by focusing on the relationship between the COM and the ZMP (Sugihara and Morisawa, 2020). Let us consider lateral motion of a humanoid as shown in Figure 1. Suppose the vertical movement of the COM and the torque about the COM are both negligibly small, the equation of motion of the COM is obtained as

$$\begin{array}{ll} \ddot{x} & =\zeta^{2}\left(x-x_{Z}\right) \end{array}$$ (1)

$$\begin{array}{ll} \zeta & \overset{def}{=}\sqrt{\frac{g}{z}} \end{array}$$ (2)

where *x* is the lateral position of the COM, *x*_Z_ is the lateral position of the ZMP, *z* is the height of the COM with respect to the nominal ground, and *g* = 9.8m/s^2^ is the acceleration due to the gravity. Note that *z* is assumed to be constant, and thus, *ζ* is also constant. The ZMP is naturally constrained within the supporting region due to the unilaterality of the contact forces as

$$x_{\text{Zmin}}\leq x_{Z}\leq x_{\text{Zmax}},$$ (3)

where *x*_Zmin_ and *x*_Zmax_ are the right and the left boundaries of the supporting region in the *x*-axis, respectively.

The COM-ZMP regulator (Sugihara, 2009) is a standing stabilization controller designed for humanoid robots, in which the desired location of the ZMP ^*d*^ *x*_*Z*_ is decided based on a piecewise-linear feedback of the COM state as

xZd=xZmax(S1:x~Z≥xZmax)x~Z(S2:xZmin<x~Z<xZmax)xZmin(S3:x~Z≤xZmin) (4)

x~Z=defxd+k1(x−xd)+k2x˙, (5)

where ^*d*^ *x* is the referential position of the COM, and *k*_1_ and *k*_2_ are feedback gains. Suppose the actual ZMP is manipulated to track the desired ZMP without delay, *i.e.*, *x*_Z_ =^*d*^ *x*_Z_. The closed-loop system becomes

x¨=ζ2x−ζ2xZmax(S1)−ζ2(k1−1)(x−xd)−ζ2k2x˙(S2)ζ2x−ζ2xZmin(S3). (6)

This is a piecewise-affine system. The gains *k*_1_ and *k*_2_ are related to the system poles − *ζq*_1_ and − *ζq*_2_ in (S2) as

$$k_{1}=q_{1}q_{2}+1,\;k_{2}=\frac{q_{1}+q_{2}}{\zeta }.$$ (7)

**FIGURE 2:**
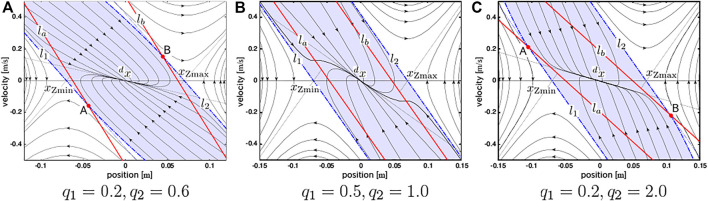
Phase portraits of the COM-ZMP model and the piecewise-linear controller with respect to different eigenvalues. **(A)** *q*_1_ = 0.2, *q*_2_ = 0.6 **(B)** *q*_1_ = 0.5, *q*_2_ = 1.0 **(C)** *q*_1_ = 0.2, *q*_2_ = 2.0.

Figure 2 shows the phase portraits of Eq. 6 with respect to some different sets of *q*_1_ and *q*_2_. Four lines that characterize the system in the figure are

$$\begin{array}{ll} & l_{1}:x+\frac{\dot{x}}{\zeta }=x_{\text{Zmin}} \end{array}$$ (8)

$$\begin{array}{ll} & l_{2}:x+\frac{\dot{x}}{\zeta }=x_{\text{Zmax}} \end{array}$$ (9)

la:xd+k1(x−xd)+k2x˙=xZmin (10)

lb:xd+k1(x−xd)+k2x˙=xZmax, (11)

where *l*_1_ and *l*_2_ are asymptotic lines in states (S1) and (S3). *l*_*a*_ and *l*_*b*_ in the portraits are the switching lines between (S1), (S2), and (S3); (S1) and (S2) are separated by *l*_*a*_, and (S2) and (S3) by *l*_*b*_, respectively. The blue areas are the stable regions, where $\left(x,\dot{x}\right)$ stably converges to (^*d*^ *x*, 0). It was also figured out that the controller with *q*_1_ = 1 satisfies the capturability condition (Pratt et al., 2006; Koolen et al., 2012), which is a sufficient condition for the standing stability (Hof, 2008).

The above controller does not assume any particular body constitution but highlights the dominant dynamics and constraint due to the unilaterality of contact forces, which is hard to be seen when focusing on only some joints.

## 3 Motion Measurement Protocol to Collect Trajectories in the State Space

**FIGURE 3:**
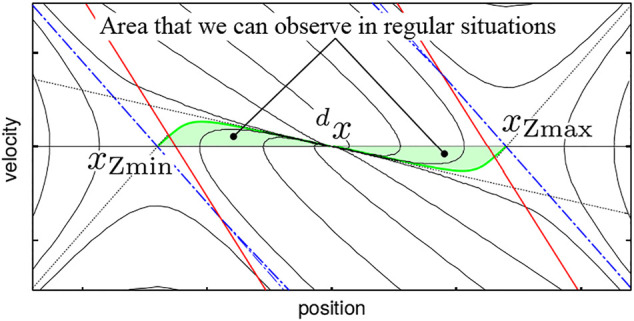
In normal situations, motion trajectories that we can observe exist within a narrow (green) area near the point of equilibrium (^*d*^ *x*, 0).

Now, we aim to see if the system represented by Eq. 6 fits the actual human behavior in stance, which is achieved by applying the system identification to measured motion data and evaluating the error of the identified parameters. It is important to collect motion trajectories from as broad area of the state space as possible for a reliable identification. However, it is not easy since a subject can start his or her motion only from a stable resting state, meaning that he or she can start motion only from points on the line *x* = 0 between *x*_Zmin_ and *x*_Zmax_. The motion trajectories that we can observe in regular situations exist within a narrow area near the point of equilibrium in the state space as depicted in Figure 3. To measure motions in a distance from the point of equilibrium, we have to devise a protocol.

We know the phase portrait in the theory as Figure 2 and can associate it with the following four typical behaviors:

A) regulatory motions to the referential position against a perturbation
B) regulatory motions from a far posture to the referential position
C) falling-down motions over the referential position
D) failure motions to recover to the referential position

**FIGURE 4:**
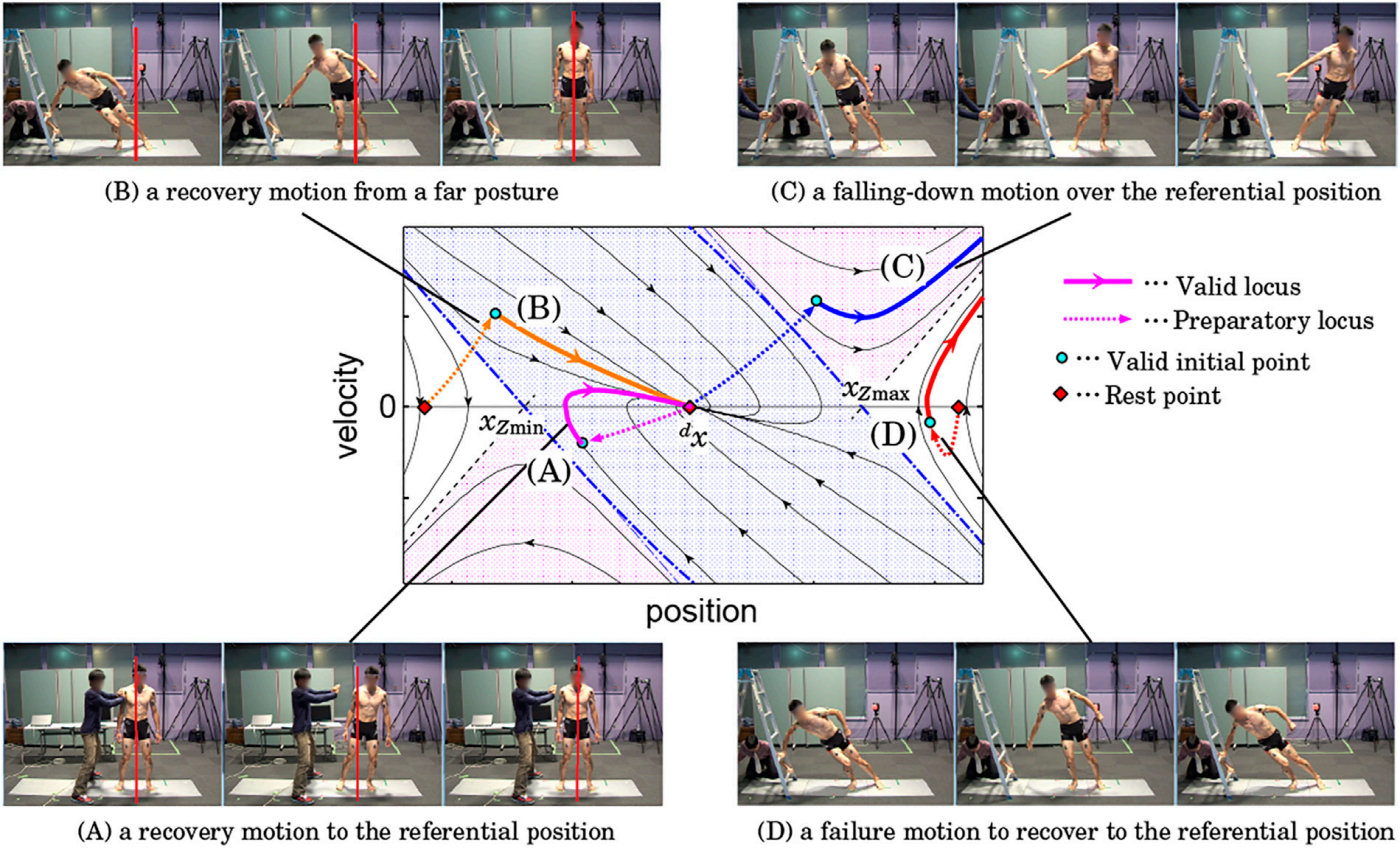
A set of motions to visualize standing stabilization behavior of a human. The labels **(A)** – **(D)** are corresponding to the groups of loci in the center figure.

Our idea is to cover the regions that correspond to the above behaviors in the state space by adding preparatory motions to each trial to accelerate the COM to the target initial states as depicted in Figure 4 with helps of a holding platform (a ladder) and an assistant person who perturbs the subject.

**FIGURE 5:**
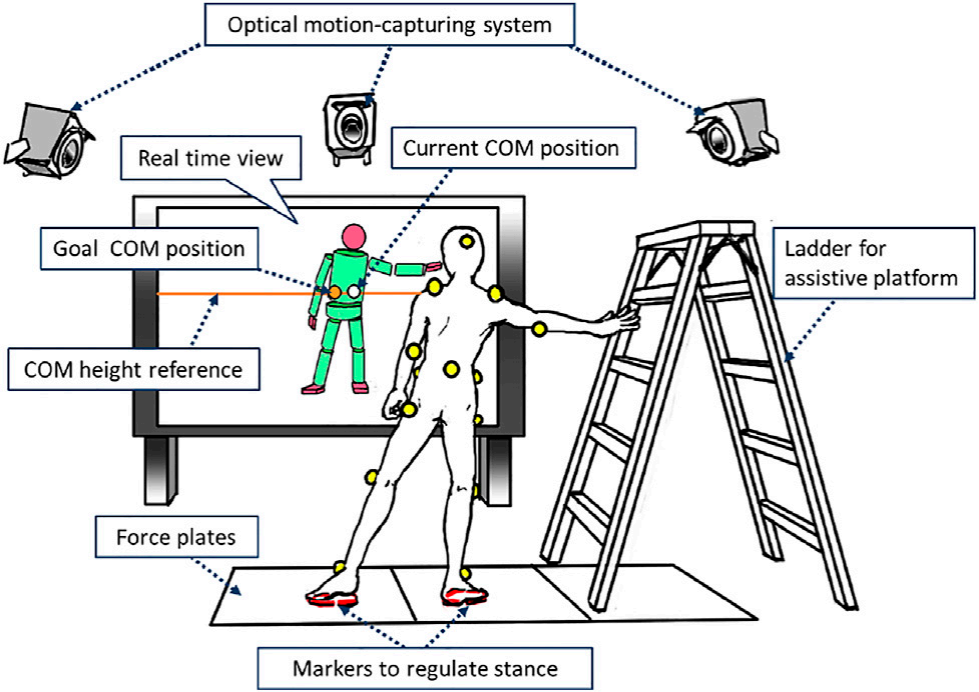
Motion measurement system setup.

We conducted motion measurement experiments based on the above protocol. The subject was a 21-year-old male, who was 181 cm tall and weighed 70 kg. His kinematics parameters and mass properties were identified before the experiments based on a method proposed by Ayusawa et al. (2011). The subject was informed about the objective and risk of the experiment and understood them in advance. Figure 5 illustrates the setup for the motion measurement. The referential point was set at the same position and visually presented to the subject on a monitor in front of him in every trial. The point was defined as the origin, i.e., ^*d*^ *x* = 0. An optical motion capture system (MAC3D System; Motion Analysis Corp.) was used to acquire 3D trajectories of retroreflective markers attached to the subject’s body every 5ms. They were converted to the trajectory of the whole-body configuration through the inverse kinematics. Then, the trajectory of the COM was computed through the forward kinematics based on the mass property identified before the experiment. Measurement noises were reduced by a second-order Butterworth filter with 2Hz of cutoff frequency. A history of the velocity and acceleration of the COM were computed by numerically differentiating it. The trajectory of the ZMP was also computed from a history of the reaction forces. The partial trajectories of preparatory motions were detected and discarded in a postprocess. In the case of (A), the phases in which the subject was pushed by the assistant person were segmented by referring the recorded scenes. Regarding the types (B), (C), and (D), distinct profiles of the reaction force from the ladder were found since the subject strongly pushed it to accelerate himself. It should be also noted that this does not require severely accurate segmentations since reliable identifications are possible if sufficient length of the trajectories is provided. In this way, 8 × 2 trajectories for the above types (A)–(D) of motions were collected in symmetric manners with respect to the point of equilibrium. Hence, the total number of trajectories was 64.

**FIGURE 6:**
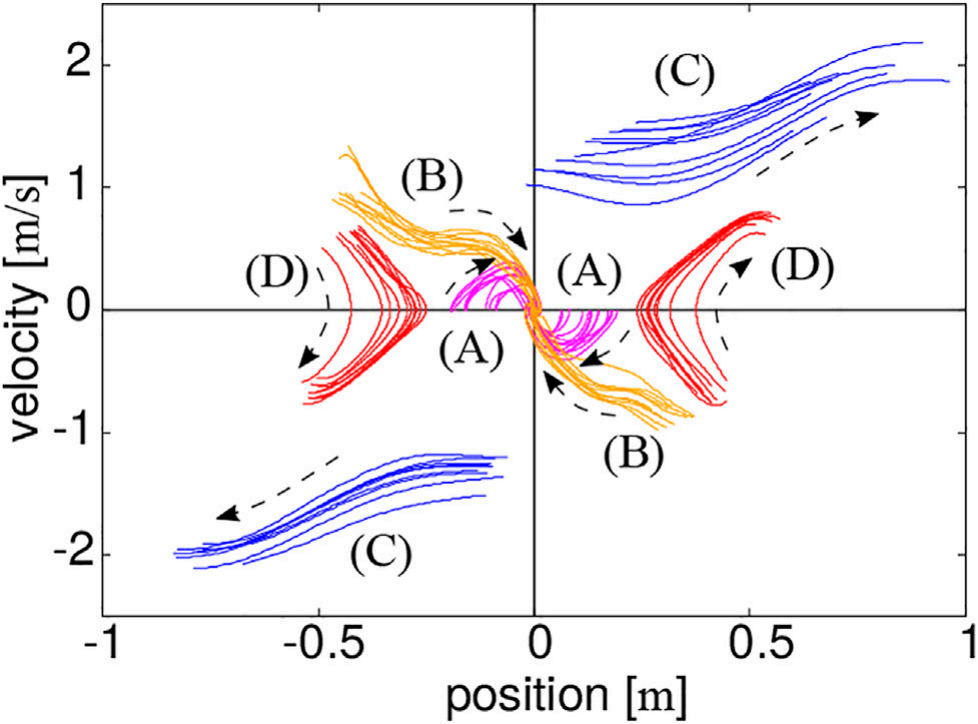
Trajectories of the COM measured based on the proposed protocol, where their directions are indicated by dashed arrows.

Figure 6 shows the trajectories of the COM reproduced based on the above-proposed protocol and plotted in the state space. The same types of trajectories are grouped by color. This qualitatively shows a similarity to the theoretical phase portrait (Figure 2).

## 4 Identification of the System Based on Clustering Techniques

Methods for the parameter identification from the measured trajectories are described in this section. Let us redescribe the system in a general form of a piecewise-affine differential equation as

$$\ddot{x}=\left\{\begin{array}{cc} c_{11}x+c_{12}\dot{x}+c_{13}\; & \left(\text{S1}\right)\\ c_{21}x+c_{22}\dot{x}+c_{23}\; & \left(\text{S2}\right)\\ c_{31}x+c_{32}\dot{x}+c_{33}\; & \left(\text{S3}\right) \end{array}\right.,$$ (12)

where {*c*_*ij*_} (*i*, *j* = 1, 2, 3) are constant coefficients. The above is equivalent to Eq. 6 if

$$\begin{array}{llllll} c_{11} & =\zeta^{2}, & c_{12} & =0, & c_{13} & =\;-\zeta^{2}x_{\text{Zmax}}, \end{array}$$ (13)

c21=−ζ2(k1−1),c22=−ζ2k2,c23=ζ2(k1−1)xd, (14)

$$\begin{array}{llllll} c_{31} & =\zeta^{2}, & c_{32} & =0, & c_{33} & =\;-\zeta^{2}x_{\text{Zmin}}. \end{array}$$ (15)

The idea here is to cluster all the samples of the discretized motion loci so that they result in the most likely identification of {*c*_*ij*_}. As shown in Eq. 4, the switching condition is whether the ZMP is inside of the supporting region or not. It may seem to be possible to know which of (S1), (S2), and (S3) the sampled state belongs to by checking the location of the measured ZMP with respect to the measured supporting region. This approach, however, does not work well since the human’s feet are neither rigid nor adhered to the ground, and the boundaries of the supporting region *x*_Zmin_ and *x*_Zmax_ are not clearly defined in the actual motions, although their nominal values are available. We examined *K*-means method (MacQueen, 1967; Duda and Hart, 1973) and EM algorithm (Dempster et al., 1977) for the clustering, the former of which also provided the initial guess of the latter.

Given a set of discretized position, velocity, and acceleration {(*x*_*k*_, *v*_*k*_, *a*_*k*_)} (*k* = 1, …, *N*) of the motion trajectories, the algorithm based on the *K*-means method is summarized as follows.

1) Divide all samples into three groups as the initial guess of {*S*_*i*_} (*i* = 1, 2, 3), where *S*_*i*_ belongs to (S*i*).
2) Identify {*c*_*ij*_} from *S*_*i*_ (*i*, *j* = 1, 2, 3) through the least square minimization.
3) Reassociate each sample {(*x*_*k*_, *v*_*k*_, *a*_*k*_)} with *S*_*i*_ such that the error $e_{ik}\overset{def}{=}|a_{k}-c_{i1}x_{k}-c_{i2}v_{k}-c_{i3}|$ is the minimum of {*e*_*jk*_} (*j* = 1, 2, 3).
4) Repeat the above 2–3 until all the samples are settled into invariant groups.

Let us name the above method (M1).

A fact that Eq. 12 forms three planes in *x*- $\dot{x}$ - $\ddot{x}$ space and the dividing lines *l*_*a*_ and *l*_*b*_ are intersections of those planes suggests another idea to alter the above step 3 to

3^'^) Find the dividing lines *l*_*a*_ and *l*_*b*_ from the tentatively identified {*c*_*ij*_}, and regroup all the samples based on them.

Let us name the above modified *K*-means method (M2).

The result of the above algorithms might be improved in terms of accuracy by conducting EM algorithm, which does not explicitly divide the samples into groups but estimates the most likely parameters through a stochastic computation. It goes with the Gaussian mixture model assumption as follows.

1) Let the result of (M1) be the initial guess of {*c*_*ij*_}, and compute the corresponding initial covariance matrices {**Σ**_*i*_} and mixing factors {*π*_*i*_}.
2) Compute the (tentative) responsibilities {*γ*_*ik*_} of each sample based on the prior probability of the corresponding sample to be produced from the current guess. (Expectation step)
3) Update {*c*_*ij*_}, {**Σ**_*i*_}, and {*π*_*i*_} from {*γ*_*ik*_}. (Maximization step)
4) Repeat the above 2–3 until the guess is settled into invariant values.

Refer to the original paper for more details. Let us name the above method (M3).

The identification based on the three methods (M1) ∼ (M3) described in the previous section was conducted with respect to the trajectories acquired in the previous section. The following techniques were additionally adopted for preprocess due to practical reasons.

• The samples {(*x*_*k*_, *v*_*k*_, *a*_*k*_)} were offset by the referential position (^*d*^ *x*, 0, 0), and each component was scaled by the minimum and maximum values of all the samples.
• Instead of Eq. 12, the following equation was assumed:

$$x=\left\{\begin{array}{cc} c_{11}^{\prime }\ddot{x}+c_{12}^{\prime }\dot{x}+c_{13}^{\prime }\; & \left(\text{S1}\right)\\ c_{21}^{\prime }\ddot{x}+c_{22}^{\prime }\dot{x}+c_{23}^{\prime }\; & \left(\text{S2}\right)\\ c_{31}^{\prime }\ddot{x}+c_{32}^{\prime }\dot{x}+c_{33}^{\prime }\; & \left(\text{S3}\right) \end{array}\right.,$$ (16)

where $\left\{c_{ij}^{\prime }\right\}$ were also constant coefficients. This coordinate transformation worked for separations of the planar clusters and was necessary for success. $\left\{c_{ij}^{\prime }\right\}$ were converted to the equivalent set {*c*_*ij*_} afterward.

• The KKZ method (Katsavounidis et al., 1994) was applied for the initialization of (M1) and (M2).

Tables 1, 2, and 3 show the identified parameters by (M1), (M2), and (M3), respectively.

**TABLE 1:** Result of parameter identification by (M1).

*i*	*c*_*i*1_	*c*_*i*2_	*c*_*i*3_
1	10.57	−0.0121	−2.321
2	−28.37	−7.842	−0.0166
3	10.09	0.411	2.014

**TABLE 2:** Result of parameter identification by (M2).

*i*	*c*_*i*1_	*c*_*i*2_	*c*_*i*3_
1	8.883	−0.360	−1.683
2	−23.32	−6.375	−0.0027
3	8.802	−0.0724	1.729

**TABLE 3:** Result of parameter identification by (M3).

*i*	*c*_*i*1_	*c*_*i*2_	*c*_*i*3_
1	9.542	−0.00057	−1.924
2	−35.92	−8.697	−0.0016
3	10.10	0.450	1.984

## 5 Discussion

We can estimate some values of *c*_11_, *c*_12_, *c*_13_, *c*_23_, *c*_31_, *c*_32,_ and *c*_33_ from nominal values of *ζ*, ^*d*^ *x*, *x*_Zmin,_ and *x*_Zmax_ before the identification based on Eqs 13–15.

• *c*_12_ ≃ 0, *c*_32_ ≃ 0
• *c*_23_ ≃ 0 since ^*d*^ *x* = 0
• *c*_11_ ≃ *c*_31_ ≃ 10 based on the nominal height of the COM
• − *c*_13_ ≃ *c*_33_ ≃ 2 based on the nominal height of the COM, the nominal width of the foot, and the nominal stance width

The results conform to the above. On the other hand, *c*_12_ and *c*_32_ in any result are different from each other by an order of magnitudes. It means that the time constants of the outward and inward falling movements are different in the case of a human. This cannot be explained when assuming a symmetric COM-ZMP model.

The standard deviations of (M1), (M2), and (M3) were 1.041, 0.764, and 1.026, respectively. Surprisingly, (M2) achieved the best result even over (M3) from this viewpoint. A possible reason is that the *K*-means method explicitly associates each sample with any of the divided regions (S1) ∼ (S3) so that the estimation accuracy is increased if the association is correct, while EM algorithm remains nonzero possibilities for all the regions which the samples are associated with.

**FIGURE 7:**
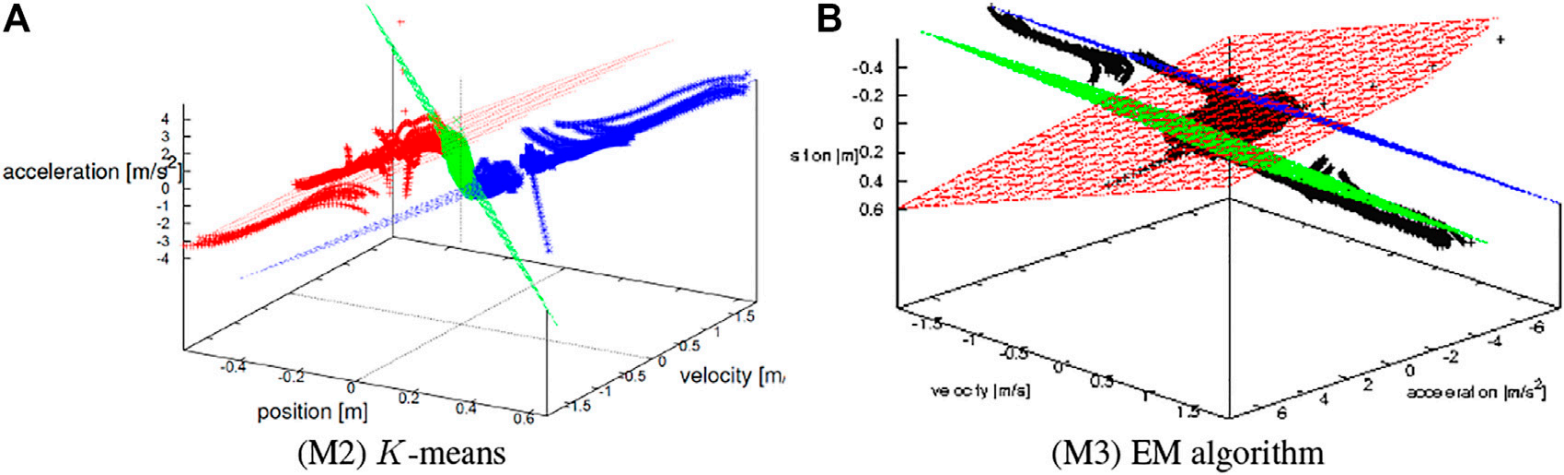
Identified planes in *x*- $\dot{x}$ - $\ddot{x}$ space. **(A)** (M2) *K*-means. **(B)** (M3) EM algorithm.

**FIGURE 8:**
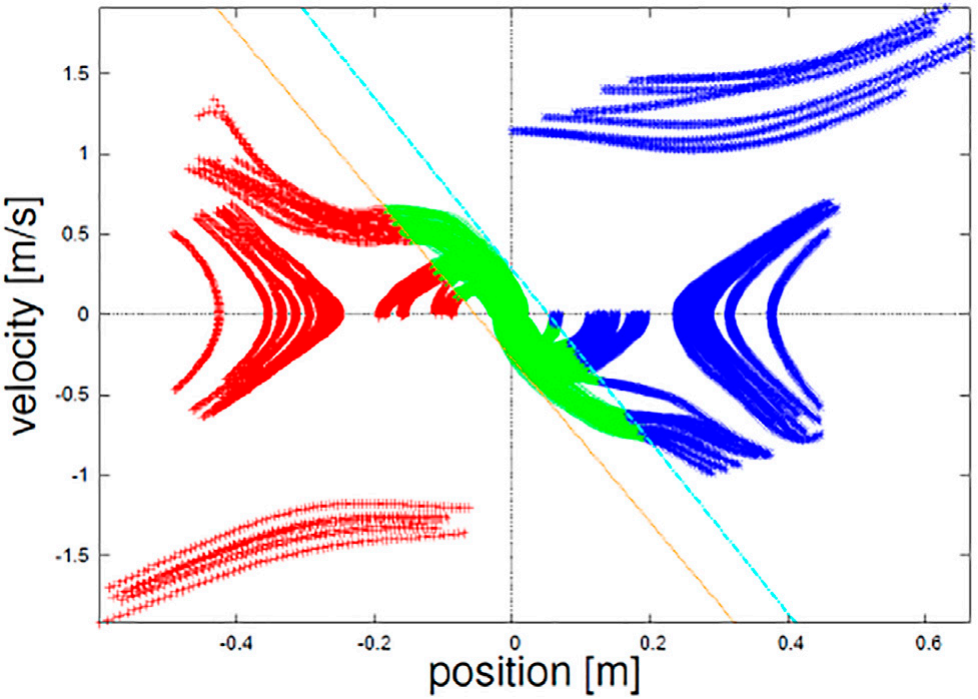
Result of division of the state space by (M2).

As noted in the previous section, Eq. 12 forms three planes in *x*- $\dot{x}$ - $\ddot{x}$ space. Figures 7A,B visualize the planes identified by (M2) and (M3), respectively. The samples in (a) are drawn in the same color with the associated plane, while those in (b) are not since EM algorithm does not explicitly associate each sample with the planes. It is confirmed that they are correctly identified in a qualitative sense and the planes in (S2) fit more than the others in particular. Figure 8 shows the top view of Figure 7A, from which it is seen that the samples are consistently grouped with respect to the dividing lines.

**FIGURE 9:**
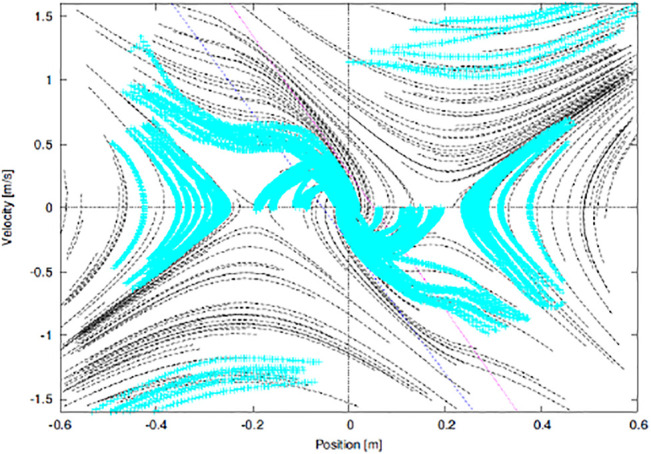
Result of the system identification by (M2).

The trajectories of the identified dynamical system by (M2) are overlaid on the measured loci in Figure 9. It seems almost consistent, though a part around the initial phase of the falling-down motion over the referential position (type (C)) is deviated from the trajectories. It might be because the human subject unconsciously pushed himself outward too much. How to control the subject’s behavior more finely or discard such preparatory trajectories should be discussed in the future.

Overall, the hypothesis that the piecewise-linear feedback scheme of the state of the COM to the manipulation of the ZMP reasonably models a human’s standing stabilization controller was supported at least with respect to the measured subject through the above discussions, although there are some points of improvement of the model.

## 6 Conclusion

An identification process of a human’s standing stabilization behavior modeled as a piecewise-linear feedback control, *i.e.*, the COM-ZMP regulator, was proposed. The contributions of this article are summarized into the following two points.

1) A protocol to collect motion data for reliable system identification was presented based on the expected phase portrait of the system.
2) Some computation methods based on clustering techniques were proposed for the identification of the piecewise-affine dynamical system.

A model of a human’s standing controller was identified by combining the above protocol and the computation method. The result quantitatively supported a hypothesis that the COM-ZMP regulator, which had been originally designed for humanoid robots, models a human’s standing control scheme. We additionally pointed out and discussed some differences of the actual human’s behavior from the model.

Note again that this study does not aim to make statistics of the identified parameters to generalize the humans’ control scheme but to develop a method to identify an individual controller. Hence, the number of subjects does not concern at the current stage, although the validity of the model should be investigated carefully by accumulating case studies.

We noticed that the studied behavior was not a “natural” but rather a “defensive” standing. We know reports of some cases (Moretto et al., 2016; Lim and Park, 2018; Neptune and Vistamehr, 2019) that humans’ behaviors are not necessarily approximated well by the COM-ZMP representation. On the other hand, we needed to pose some conditions on the subject to control his behavior for reliable identification as the first step. We need to relax the conditions by extending the model to elaborate more natural human behaviors that require hip-strategy-like counter-phase coordination of joints. Such a model may be retransferred to a whole-body control scheme for humanoid robots. This is one of the important future works.

The parameters of controllers for humanoid robots are designed in general based on the stabilization performance and the hardware responsivity against perturbations. We think that this knowledge is directly utilized in an evaluation of humans’ body control abilities. Namely, the abilities to coordinate the whole body, which are hardly seen in a single physical performance such as the muscle strength and the lung capacity, can be measured based on the identified parameters as well as other holistic approaches (Torricelli et al., 2020).

## Data Availability

The datasets presented in this article are not readily available because it includes personal information. Requests to access the datasets should be directed to zhidao@ieee.org.
